# Towards the Antiviral Agents and Nanotechnology-Enabled Approaches Against Parvovirus B19

**DOI:** 10.3389/fcimb.2022.916012

**Published:** 2022-06-20

**Authors:** Xi Hu, Chen Jia, Jianyong Wu, Jian Zhang, Zhijie Jiang, Kuifen Ma

**Affiliations:** ^1^Department of Clinical Pharmacy, The First Affiliated Hospital, Zhejiang University School of Medicine, Hangzhou, China; ^2^Zhejiang Provincial Key Laboratory for Drug Evaluation and Clinical Research, The First Affiliated Hospital, Zhejiang University School of Medicine, Hangzhou, China; ^3^Department of Pharmacy, Lanzhou University Second Hospital, Lanzhou, China; ^4^Kidney Disease Center, The First Affiliated Hospital, Zhejiang University School of Medicine, Hangzhou, China

**Keywords:** parvovirus B19, life cycle, antiviral agents, nanotechnology, therapeutic strategy

## Abstract

Parvovirus B19 (B19V) as a human pathogenic virus, would cause a wide range of clinical manifestations. Besides the supportive and symptomatic treatments, the only FDA-approved antiviral drug for the treatment of B19V is intravenous immunoglobulins, which however, have limited efficacy and high cost. By far, there are still no virus-specific therapeutics clinically available to treat B19V infection. Therefore, exploiting the potential targets with a deep understanding of the life cycle of B19V, are pivotal to the development of B19V-tailored effective antiviral approaches. This review will introduce antiviral agents *via* blocking viral invasion, inhibiting the enzymes or regulatory proteins involved in DNA synthesis, and so on. Moreover, nanotechnology-enabled approaches against B19V will also be outlined and discussed through a multidisciplinary perspective involving virology, nanotechnology, medicine, pharmaceutics, chemistry, materials science, and other fields. Lastly, the prospects of the antiviral agents and nanosystems in terms of fabrication, clinical translation and potential breakthroughs will be briefly discussed.

## Introduction

Human parvovirus B19 (B19V) is a single-stranded DNA (ssDNA) virus in the family Parvoviridae (Luo and Qiu, 2015). During the last few years, B19V has been recognized as the causative agent of wide ranges of diseases, such as pure red cell aplasia (PRCA), inflammatory cardiomyopathy, systemic autoimmunity, and so on (Heegaard and Brown, 2002; Abdelrahman et al., 2021). It causes diverse clinical manifestations ranging from asymptomatic or mild to more severe outcomes, depending on the interplay between the viral properties as well as the physiological and immune status of the infected individuals. Indeed, in many clinical settings, there would be adverse clinical outcomes without intervention, especially in immunosuppressed patients such as solid-organ-transplant recipients (Eid et al., 2006). For immunocompromised patients, PRCA resulting from the infection of erythroid progenitors cells (EPCs) is one of the most common clinical manifestation of B19V infection (Inoue et al., 2021). Intravenous immunoglobulins (IVIG) is the only available option for the treatment of B19V infection in case of chronic infections or more rarely severe acute infections in the impaired immune system (Mouthon et al., 2005; Crabol et al., 2012). However, its high cost cannot be ignored and symptoms often recur when IVIG treatment is interrupted (Ogawa et al., 2008). Therefore, exploring specific anti-B19V agents and effective therapeutic treatments is considered of intense importance.

This review will focus on the life cycle of B19V and effective antiviral agents, aiming at providing a comprehensive overview of the rational fabrication of B19V-tailored approaches. Firstly, the life cycle of B19V and related potential therapeutic targets will be discussed, laying the foundation for exploiting the potential therapeutic approaches. Moreover, based on the above analyses, the antiviral agents will be classified into blocking viral invasion, inhibiting the enzymes or regulatory proteins involved in DNA synthesis, and so on, and clearly elaborated. Furthermore, nanotechnology-enabled approaches against B19V will be outlined and discussed through a multidisciplinary perspective encompassing virology, nanotechnology, medicine, pharmaceutics, chemistry, materials science, and other fields. Lastly, the prospects of the antiviral agents and nanosystems in terms of fabrication, clinical translation and potential breakthroughs will be briefly discussed, in order to speed up the development of antiviral agents and nanotechnology-enabled approaches against B19V.

## Virus Structure, Life Cycle and Virus-Cell Interaction of B19V

The genome of B19V is a 5.6kb linear ssDNA, consisting of a unique internal region with all the coding sequences (Qiu et al., 2017). Wherein, B19V genome contains a 67-bp long minimum origin of replication (Ori) with the same and reverse inverted terminal repeats (ITRs) at each ends (Guan et al., 2009). Ori contains ITRs, signal transducers and activators of transduction 5 (STAT5) binding site, non-structural proteins 1 (NS1) binding elements (NSBE) and potential host factor binding sites (Sanchez et al., 2016; Ganaie et al., 2017). The unique internal region encodes three main kinds of proteins including colinear capsid proteins (VP1 and VP2), NS1, and several other minor non-structural proteins (7.5 kDa, 9 kDa and 11 kDa). The capsid that is composed of 5-10% VP1 (minor capsid protein) and 90-95% VP2 proteins (main capsid protein), forms an icosahedral structure with T=1 arrangement and approximately 23-26 nm in diameter, and is crucial for viral DNA encapsidation (Gallinella, 2018). Besides, nonstructural proteins (i.e., phosphoproteins) are associated with efficient viral replication, gene transcription, packaging and release of infectious viral particles (Ganaie and Qiu, 2018).

**Figure 1:**
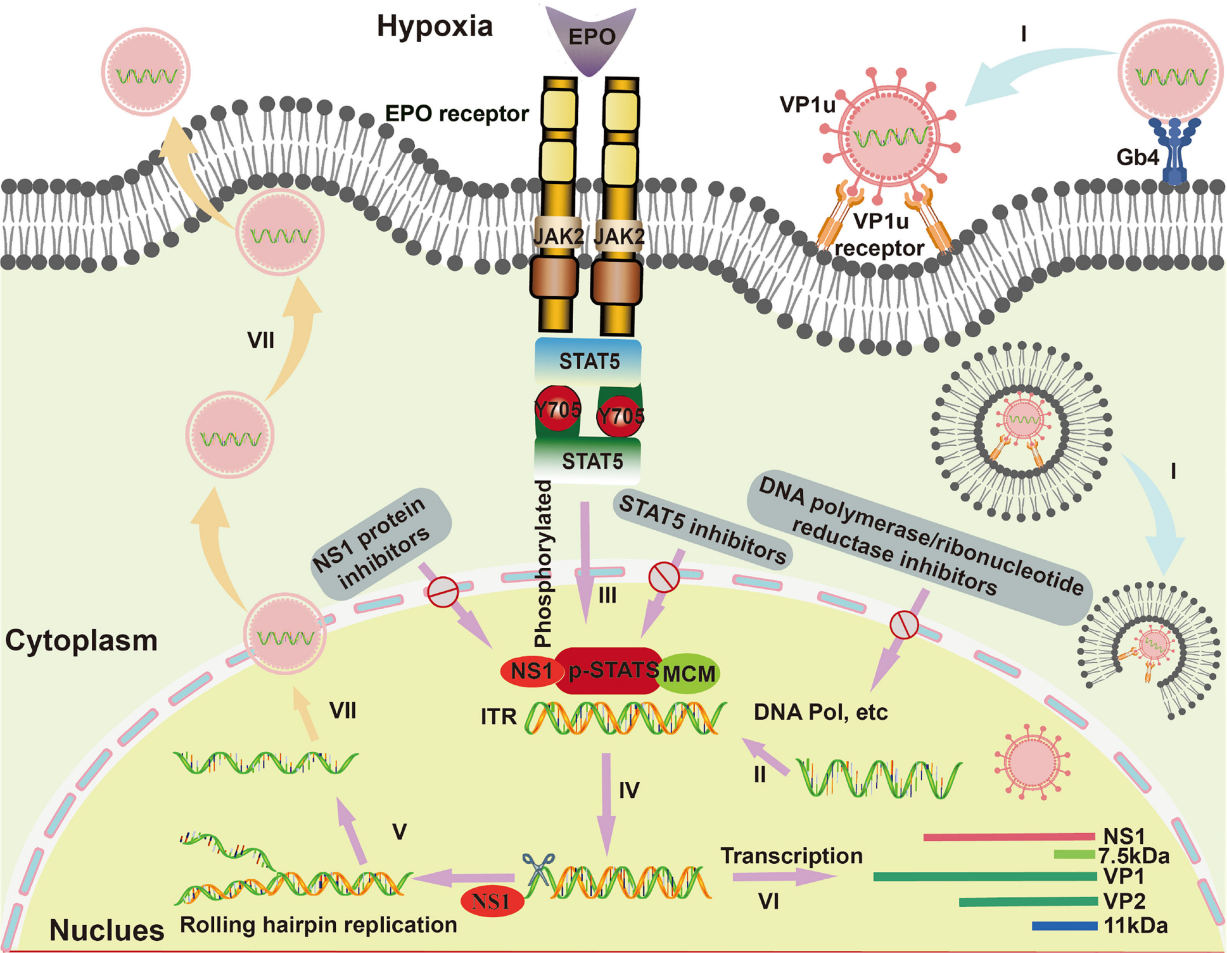
The life cycle and virus-host cell interactions of B19V in EPCs. I. B19V binds to membrane glycosphingolipid globoside (Gb4), virion uncoats and releases the ssDNA genome. II. Virion synthesizes the second strand form inverted terminal repeats (ITRs) under the action of cellular DNA polymerase and so on. III. Erythropoietin (EPO) and hypoxia activate and increase the phospho-signal transducers and activators of transcription (pSTAT5), which interacts with minichromosome maintenance (MCM) complex for initiation of viral DNA replication by a rolling hairpin model. Then, the non-structural proteins 1 (NS1) protein binds to the NS1 binding elements (NSBE) site, where pSTAT5 recruits the MCM complex and nicks one of the single strands. IV. An open-ended double-stranded DNA intermediate is formed under the action of the NS1 ATP helicase function. V. The double-stranded ITRs intermediate initiates a new round of viral genomic DNA replication. VI. Regarding the transcription of B19V, mRNA encodes the NS1 protein, VP1/2 protein and 11 kDa protein. VII. Capsids and single-stranded genomes assemble into virions, which are ultimately released from host cells.

The virus-host cell interactions of B19V are illustrated in **Figure 1**. Since the main receptor of B19V is the neutral glycosphingolipid globoside (Gb4, also known as P antigen) on the surface of the EPCs, B19V shows a strong tropism for human EPCs (Manaresi and Gallinella, 2019). Moreover, B19V would elicit a complex set of effects in the infected EPCs, including inducing cell apoptosis and cell cycle arrest, as well as human erythrotopenia and anemia (Xu et al., 2017). The binding of B19V capsid to membrane Gb4 is the first step for cell infection, then triggering the conformational changes to expose the VP1 unique region (VP1u) for binding an unknown co-receptor (Leisi et al., 2016a, Leisi et al., 2016b). After endocytosis, B19V would escape from lysosomal degradation and transfer to cell nucleus (Caliaro et al., 2019). Inside the nucleus, the B19V virion uncoats and releases the ssDNA genome, followed by a series of macromolecular synthesis (e.g., DNA replication, RNA synthesis (transcription), and protein synthesis (translation)) to trigger a replicative cycle (Luo and Qiu, 2015; Ganaie and Qiu, 2018).

The B19V replication is involved in double-stranded (dsDNA) formation and rolling hairpin replication. Firstly, the negative-sense ssDNA of the viral genome generates complementary positive-sense DNA with the ITRs as a primer under the action of cellular DNA polymerase, serving as a template for DNA replication and transcription (Cotmore et al., 2014). Indeed, since B19V does not encode DNA polymerase, its genome replication is entirely dependent on host cells (Luo and Qiu, 2015), where their DNA polymerase δ (Pol δ) and Pol α are essential for viral replication (Zou et al., 2018). Besides, B19V infection induces cell cycle arrest at S phase, in which various S phase replication factors such as proliferating cell nuclear antigen (PCNA), replication factor C (RFC), the minichromosome maintenance (MCM) complex are actively recruited to viral DNA replication centers (Zou et al., 2018). In addition, it has been reported that B19V replication is critically dependent on erythropoietin (EPO) and hypoxia (Chen et al., 2011); thereinto, EPO could activate cellular signal transduction under hypoxic condition and then phosphorylate STAT5 protein. The phosphorylated STAT5 protein (pSTAT5) could specifically bind to STAT5 binding sites in the Ori region, and interact with MCM complex for initiation of viral DNA replication by a rolling hairpin model (Sanchez et al., 2016; Ganaie et al., 2017). In the process of rolling hairpin replication, the NS1 protein would: 1) bind to the NSBE site, where pSTAT5 recruits the MCM complex, 2) nick one of the single strands, 3) complete the annealing extension of the ITRs under the action of the NS1 ATP helicase function, 4) form an open-ended double-stranded DNA intermediate (Burnett et al., 2006; Guan et al., 2009). The intermediate can be used as a new primer to initiate a new round of viral genomic DNA replication (Burnett et al., 2006; Guan et al., 2009; Tewary et al., 2014). As to the transcription of B19V, mRNA encodes the NS1 protein in the early phase, while constructs coding for structural VP1/2 proteins and 11 kDa proteins in the later phase (Manaresi and Gallinella, 2019). Moreover, the accumulation of VP1/2 proteins leads to the assembly of capsids for the encapsidation of progeny single-stranded genomes and then the formation of virions, which are consequently released from infected cells through inducing cell lysis or apoptosis (Manaresi and Gallinella, 2019).

## Antiviral Agents

B19V infection can cause significant morbidity especially in immunosuppressed patients, such as solid-organ-transplant recipients (Eid et al., 2006). Up till now, the treatment of B19V is mainly involved in symptomatic or supportive therapy, in particular blood transfusion that is required to overcome acute or chronic anemia (Crabol et al., 2012). However, by far, there are still no virus-specific therapeutics clinically available towards B19V infection. Therefore, there is an urgent need to explore B19V-specific therapeutics in both laboratory and clinical studies. Based on the above analysis about life cycle and virus-host cell interactions of B19V, the antiviral agents will be classified into blocking viral invasion, inhibiting the enzymes or regulatory proteins involved in DNA synthesis, and so on, for further clear elaboration.

### Viral Invasion Inhibition

Immunoglobulins and monoclonal antibodies have been developed to inhibit viral invasion by preventing viral attachment to the cellular receptors. IVIG is the only available option for the treatment of B19V infection in case of persistent infections or more rarely severe acute infections in the impaired immune system (Mouthon et al., 2005; Crabol et al., 2012). It prevents the infectivity of B19V possibly by directly binding to functionally relevant epitopes on the viral capsid as well as immune modulatory mechanisms (Chaigne and Mouthon, 2017; Manaresi and Gallinella, 2019). However, the high cost cannot be ignored and symptoms often recur when IVIG treatment is interrupted (Ogawa et al., 2008).

To explore the alternative to IVIG, Gigler et al. studied the potent neutralizing activity of human immunoglobulin G monoclonal antibodies (MAbs), which are generated from individuals suffering from persistent B19V infection. These MAbs have proved to be the important therapeutic reagents for B19V-infected pregnant women or chronically infected patients (Gigler et al., 1999). Additionally, Pei et al. obtained canine immunoglobulin F(ab′)_2_ fragments from inactivated-feline parvovirus virus (FPV)-immune canine *via* extracting IgG, pepsin digestion and purification. Interestingly, anti-FPV-specific F(ab′)_2_ fragments have showed efficient neutralizing activity against FPV *in vitro*, and could significantly alleviate the clinical symptoms and reduce the viral loads in the intestinal tract of FPV-infected cats (Pei et al., 2020). These studies might become an available option and provide inspiration for the B19V vaccine development.

### Inhibitor of Enzymes Involved in DNA Synthesis

DNA polymerase inhibitors (e.g., nucleotide analogues, pyrophosphate analogues), and ribonucleotide reductase inhibitors (e.g., hydroxyurea) have been developed to directly target the viruses and interfere with DNA synthesis of B19V.

#### Nucleotide Analogues

The replication of B19V depends on cellular DNA polymerase that is essential for dsDNA replicative intermediate formation, and thus is potentially inhibited by the nucleotide analogues (Manaresi and Gallinella, 2019). The nucleotide analogues (e.g., cidofovir, brincidofovir, telbivudine, and tenofovir) as the selective inhibitors of DNA polymerase, inhibit viral DNA synthesis and thereby suppress viral replication (Kim et al., 2006; Murphy and Valentovic, 2017; Manaresi and Gallinella, 2019).

As for cidofovir, Bonvicini et al. studied its effect against B19V in both UT7/EpoS1 cell line and EPCs that are generated from peripheral blood mononuclear cells. Cidofovir showed a relevant inhibitory activity on B19V replication within infected UT7/EpoS1; while in EPCs, it provoked a significant reduction in B19V DNA amounts (68.2–92.8%) and the viral infectivity after being released from EPCs at the concentration of 500 μM (Bonvicini et al., 2015). Besides, they further found cidofovir-induced B19V replication inhibition could be further enhanced by extending exposure time following infection (Bonvicini et al., 2016). Moreover, Nair et al. reported a case of an 18-year-old male with collapsing glomerulopathy (CG) associated with B19V infection, who was treated by IVIG while rapidly progressed to end-stage kidney disease. After combining with cidofovir, B19V viremia was slowly cleared, thus allowing him receiving kidney transplant and minimizing the risk of recurrent CG (Nair et al., 2020). Although the positive inhibitory effect of cidofovir towards B19V replication, some shortcomings including the need of high concentration and extended exposure time in primary EPCs *in vitro*, as well as the non-neglectable toxicity prevent the widespread use in the effective treatment of B19V.

Furthermore, Bua et al. compared the antiviral activity of brincidofovir (a lipid conjugated prodrug of cidofovir) and cidofovir against B19V. Brincidofovir showed much lower EC 50 values (0.22–0.63 μM in UT7/EpoS1 cells, 6.6–14.3 μM in EPCs) than cidofovir (16.1 μM in UT7/EpoS1 cells, > 300 μM in EPCs) (Bua et al., 2019). The antiviral activity of brincidofovir and cidofovir is directly correlated to cidofovir-diphosphate, which acts as an alternate substrate for viral DNA synthesis (Magee and Evans, 2012).

Telbivudine, a thymidine analogue for widely treating chronic hepatitis B, possesses antiviral and anti-inflammatory properties (Kim et al., 2006). Linthout et al. evaluated the endothelial-protective potential of telbivudine in B19V-infected human microvascular endothelial cells-1. Telbivudine (10 ng/mL) decreased the apoptosis, endothelial-to-mesenchymal transition, the expression of transforming growth factor-β1 and of tenascin-C, and the oxidative stress of B19V-induced endothelial cell. Moreover, telbivudine improved chronic lymphocytic myocarditis patients with B19V transcriptional activity in a single-use approach, whereas its effectivity should be further proven in clinical placebo-controlled studies (Van Linthout et al., 2018). Additionally, Zobel et al. found telbivudine could reverse B19V-induced dysregulation of BIRC3 in early outgrowth EPCs and endothelial colony-forming cells (ECFC), and thus intervene in the apoptosis pathway and protect susceptible cells from death. Therefore, telbivudine might be an effective treatment option for B19V *via* protecting the host cell from apoptosis (Zobel et al., 2019).

Regarding tenofovir, Koenig et al. reported a 19-year-old female patient suffering from a fulminant B19V myocarditis after chemotherapy, was completely recovered by tenofovir disoproxil treatment (245 mg once daily for 6 months) in combination with guideline-recommended heart failure therapy. It demonstrated tenofovir disoproxil is a safe and effective antiviral drug for B19V-induced acute myocarditis (Koenig et al., 2022).

Therefore, nucleotide analogues have revealed the effective inhibition of viral DNA synthesis for the treatment of B19V. However, their inhibitory efficiency and biosafety should be further ameliorated for the widespread use in the treatment of B19V infection.

#### Pyrophosphate Analogue

Foscarnet is an organic analogue of inorganic pyrophosphate for inhibiting viral replication (Zarrouk et al., 2021). Yu et al. studied foscarnet therapy for kidney transplantation recipients with parvovirus B19V-associated PRCA (B19V-PRCA). Among 11 patients who received foscarnet therapy, 10 patients responded well to foscarnet with increased mean hemoglobin level (from 68.5 ± 9.3 g/L to 73.2 ± 8.8 g/L) and the mean percentage of reticulocytes (from 0.1 ± 0.0% to 7.6 ± 2.9%), and the decrease of median serum genome copy number of B19V. Therefore, foscarnet might be an alternative option of IVIG for B19V-PRCA in kidney transplantation recipients (Yu et al., 2021).

#### Inhibitor of Ribonucleotide Reductase

Hydroxyurea is an S phase inhibitor of DNA synthesis *via* targeting cellular ribonucleotide reductase enzyme (Xu et al., 2016). Bonvicini et al. reported hydroxyurea could not only inhibit B19V replication in UT7/EpoS1 and EPCs (EC_50_ values: 96.2 µM and 147.1 µM, respectively), but also reduce cellular DNA replication *via* the cytostatic effect. In this process, hydroxyurea would prevent infected or uninfected cells from the G1/S phases to G2/M, and arrest with a 2N DNA content to interfere B19V active replication (Bonvicini et al., 2017).

Moreover, sickle cell disease (SCD) is a typical situation where B19V infection exerts profound pathological effects, for example, causing transient aplastic crisis, and often requires hospitalization and intense supportive therapy (Obeid Mohamed et al., 2019). Therefore, the drug with dual effects on the course of the underlying disease and B19V infection would be beneficial to the treatment. In treated SCD patients, hydroxyurea can reach peak plasma concentrations of 250–400 mM (McGann and Ware, 2015), ensuring the efficient suppression of B19V replication as studied *in vitro*. More importantly, Hankins et al. found hydroxyurea may reduce the requirements for blood transfusion and attenuate the symptoms during transient aplastic crisis episodes in children with SCD caused by B19V infection in a clinical investigation. In addition, hydroxyurea might also be associated with a prolonged lifespan of erythrocytes (Hankins et al., 2016).

### Inhibitor of Regulatory Proteins Involved in DNA Synthesis

Inhibitors of the regulatory proteins involved in DNA synthesis, for instance, STAT5 inhibitors (e.g., pimozide) and NS1 protein inhibitors, are feasible to suppress the replication of B19V viral genome for the treatment of B19V infection.

#### STAT5 Inhibitors

Since STAT5 phosphorylation is essential for DNA replication of B19V (Ganaie and Qiu, 2018), pimozide as a FDA-approved antipsychotic drug could dephosphorylate STAT5 and thus inhibit B19V replication (Ganaie et al., 2017). Pimozide has an EC_50_ of 2.7 ± 0.69 μM in inhibition of B19V infection in *ex vivo* expanded EPCs (Ganaie et al., 2017). Besides, the development of other STAT5 inhibitors is also essential to combat B19V infection.

#### NS1 Protein Inhibitors

**Figure 2:**
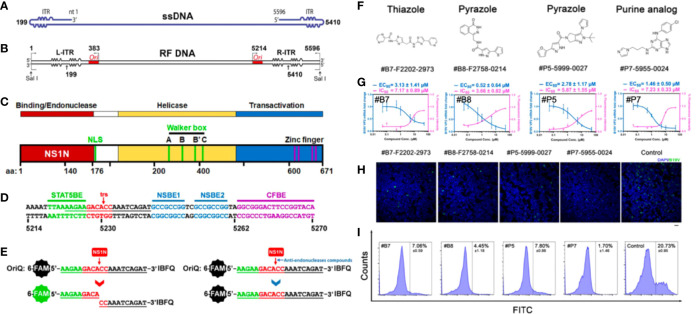
Fluorophore-based *in vitro* nicking assay for high throughput screening of anti-B19V compounds. **(A)** B19V ssDNA genome. **(B)** B19V RF DNA. **(C)** A diagram of B19V NS1 protein. **(D)** Minimal replication origin (Ori) of B19V. **(E)** Diagram of fluorophore-based *in vitro* nicking assay for high throughput screening of anti-B19V antivirals. **(F–I)** Four selected compounds (#B7 (F2202-2973), #B8 (F2758-0214), #P5 (F5999-0027) and #P7 (F5955-0024)) for inhibition assay in CD36^+^ EPCs. **(F)** Chemical structures. **(G)** Measured EC_50_ and IC_50_. **(H)** Immunofluorescent assay (green, anti-B19V capsid antibody). **(I)** Flow cytometry of EPCs stained with a mouse anti-B19V capsid monoclonal antibody and a FITC conjugated secondary antibody. Reproduced with permission (Ning et al., 2022). Copyright ^©^ 2022 American Society for Microbiology.

B19V replication follows a rolling-hairpin model, in which the large nonstructural protein NS1 introduces a site-specific single-strand nick in the origins of viral DNA replication, and NS1 performs endonuclease activity *via* the N-terminal initiation binding domain (Luo and Qiu, 2015). Cutting off the origin of viral replication origin is a key step in rolling hairpin-dependent viral DNA replication. Xu et al. developed a fluorophore-based *in vitro* nicking assay of the replication origin using the origin-binding domain of NS1 for high-throughput screening of small-molecule compounds as anti-B19V drug candidates. They screened 96 small-molecule compounds and identified 8 compounds that inhibited NS1 nicking of the B19V Ori *via* an *in vitro* nicking assay. And among these, three compounds with a similar flavonoid chemical structure inhibited the B19V DNA replication in UT7/Epo-S1 cells (Xu et al., 2019). They further screened 17,040 compounds based on the above fluorophore-based *in vitro* nicking assay, and identified 84 compounds to inhibit nicking in a dose-dependent manner. Among them, four potential compounds (i.e., #P5, #P7, #B7, #B8) were further studied and demonstrated evident inhibition of B19V replication in CD36^+^ EPCs. And the purine derivative P7 (EC_50_, 1.46 μM; CC_50_, 71.8 μM) and the pyrazole derivative B8 (EC_50_, 0.52 μM; CC_50_, 66.1 μM) apparently outcompeted some reported active identities (e.g., cidofovir, brincidofovir, flavonoid, and pimozide) in inhibition of B19V infection in EPCs (**Figure 2**) (Ning et al., 2022). However, the safety and efficacy of these compounds should be further evaluated in preclinical and clinical trials.

### Others

Regarding to some other broad spectrum antiviral agents, for example, coumarin not only inhibits many proteins involved in the transcription/translation in virus life cycle, but also modulates the host cell signaling pathways for blocking the virus replication (Mishra et al., 2020). Moreover, it has received a considerable attention as a scaffold structure for designing novel orally non-peptidic antiviral agents (Hassan et al., 2016). Conti et al. synthesized a series of 3-(imidazo[2,1-b]thiazol-6-yl)-2H-chromen-2-one derivatives based on the coumarin scaffold structure. Through studying structure-activity relationship in UT7/EpoS1 and EPCs, they found some compounds showed predominant inhibitory activity on both cell viability and viral replication (Conti et al., 2019).

Despite the above outcomes, proactive efforts towards the development of anti-B19V agents are still urgently needed, which selectively interfere with the virus without damaging the host’s cells at low drug doses. Regarding the inhibitors of enzymes and/or regulatory proteins involved in DNA synthesis, their effectiveness and selectivity is still limited according to these research results. On the one hand, we should focus on modifying the chemical structures or developing nanomedicines to improve the selectivity index and attenuate the side effects. On the other hand, other drug targets such as uncoating inhibitors and protease inhibitors can also been explored and evaluated for the treatment of B19V infection.

## Nanosystems

Nanotechnology is not only inspired by virology to develop novel therapeutics and vaccines, but also at the forefront in combatting dangerous viruses (Reis et al., 2020; Hu et al., 2021; An et al., 2021; Lee et al., 2022). However, up to date, there is still a long way to go to develop B19V-tailored nanosystems. Some anti-B19V agents-encapsulated nanosystems and antiviral inorganic nanosystems have been developed in the last few years, while their anti-B19V efficacy should be further verified. Besides, biotherapeutic and biomimetic nanosystems that have been emerging as an attractive paradigm for antiviral therapeutics and vaccine development, could also be explored to specifically prevent and treat B19V infection in the future. Here, we try to present the design and fabrication strategies of B19V-tailored nanosystems, and offer some suggestions for its nanotechnology-based therapeutics and vaccine development.

### Antiviral Agents-Encapsulated Nanosystems

Drug delivery nanosystems incorporating antiviral agents embody the advantages in prolonging blood circulation time, improving cellular selectivity and controlled release, and consequently improving antiviral effects compared to free antiviral agents (Cojocaru et al., 2020; Chen et al., 2021). And nanosystems that encapsulate anti-B19V agents would pave the way to the nanotechnology-aided B19V treatment.

Regarding the cell-proliferation inhibitor hydroxyurea with amino groups, some drug delivery nanosystems based on organic and/or inorganic nanomaterials have been developed. For example, Tazhbayev et al. loaded hydroxyurea into human serum albumin (HSA) nanoparticles (NPs) by crosslinking HSA *via* urea and cysteine. The HSA-hydroxyurea-NPs had a spherical morphology with an average size of less than 200 nm and encapsulation efficiency of ~77% (Tazhbayev et al., 2019). Moreover, Chaghervand et al. prepared functionalized Fe_3_O_4_/SiO_2_-NH_2_ NPs with methoxy-PEGylated chitosan (Cs-g-mPEG_2000_) to load hydroxyurea *via* electrostatic adsorption (Chaghervand et al., 2022).

**Figure 3:**
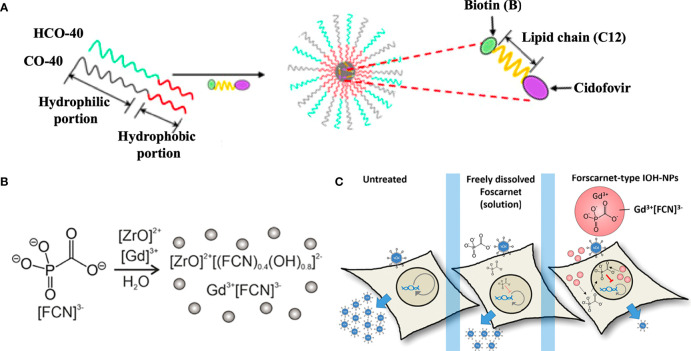
Antiviral agents-encapsulated nanosystems. **(A)** Fabrication of a biotinylated lipid prodrug of cyclic cidofovir (B-C12-cCDF) within polymeric micelles. Reproduced with permission (Mandal et al., 2017). Copyright ^©^ 2017 American Chemical Society. **(B)** Fabrication of foscarnet (FCN)-, ZrO2+- and Gd3+-contained inorganic-organic hybrid NPs (IOH-NPs). **(C)** FCN-type IOH-NPs presented the promising antiviral activity for the delivery of antivirals against viruses. Reproduced with permission (Khorenko et al., 2022). Copyright ^©^ 2022, American Chemical Society.

As to nucleotide analogues including cidofovir, brincidofovir, telbivudine, and tenofovir, there are also various nanosystems developed in recent decades. Actually, the majority of nucleotide analogues are hydrophilic and thus possess a relatively low affinity for hydrophobic block of polymers, thus it is hard to encapsulate them into NPs *via* hydrophobic interaction. Varan et al. loaded cidofovir into the poly(ethylene glycol)-polycaprolactone (PEG-PCL) NPs using the W/O/W emulsion technique (Varan et al., 2019). Moreover, the prodrug strategy by conjugation to a hydrophobic molecule could convert the hydrophobic drug into a lipophilic prodrug for enhanced encapsulation and biodistribution *in vivo* (Gautam et al., 2021). For instance, Mandal et al. formulated a biotinylated lipid prodrug of cyclic cidofovir (B-C12-cCDF) within polymeric micelles by using polyoxyethylene hydrogenated castor oil 40 (HCO-40) and octoxynol 40 (OC-40) (**Figure 3A**) (Mandal et al., 2017). Therefore, numerous pharmaceutical formulation strategies and chemical strategies could be utilized to develop promising antiviral drug delivery nanosystems, which is worthy of wide attention for anti-B19V therapy.

Additionally, foscarnet-encapsulated nanosystems have also been developed for antiviral treatment. For instance, Russo et al. prepared a ionotropic gelation of chitosan induced by foscarnet, which served as an ionotropic agent in a manner similar to tripolyphosphate anion. The foscarnet-chitosan crosslinked NPs showed a controlled drug release and maintained the antiviral activity *in vitro* (Russo et al., 2014). Moreover, Khorenko et al. fabricated a foscarnet-, ZrO2+- and Gd3+-contained inorganic-organic hybrid NPs (IOH-NPs) as a saline antiviral nanocarrier (**Figure 3B**), which possessed a small size (20−30 nm), high biocompatibility, and high drug loading (up to 44 wt %). The antiviral activity of the foscarnet-type IOH-NPs significantly outperformed that of free foscarnet at the level of clinical formulations (**Figure 3C**) (Khorenko et al., 2022).

Although the feasible design and fabrication, the antiviral potency of these drug delivery nanosystems against B19V both *in vitro* and *in vivo* needs to be clearly evaluated. Moreover, the combination of different types of antiviral agents may be worth further research as well for the synergetic treatment of B19V infection.

### Antiviral Inorganic Nanosystems

In recent decades, different types of metal-based NPs have shown antiviral properties (Lee et al., 2022). Metal-based NPs, such as silver (Ag), selenium (Se), titanium dioxide (TiO_2_), graphene oxide (GO), and so on, can be developed to inhibit the infection of virus in cells or directly inactivate the viruses by releasing toxic ions, generating reactive oxygen species, and/or eliciting photo-based reactions (e.g., photothermal reaction) (Kerry et al., 2019; Talebian et al., 2020). Among these, from the perspective of chemical synthesis and viral life cycle, there are several effective chemical strategies, for instance, tuning chemical composition, size as well as surface chemistry of metal-based NPs, to remarkably improve the antiviral effects.

#### Chemical Composition

AgNPs reveal the highly great antiviral efficacy against different types of viruses (Aschalew et al., 2016; Park et al., 2018). The antiviral mechanisms of AgNPs might involve several processes: 1) interact with the viral surface to destruct the viral genomic material or prevent it from penetrating the cell membrane; 2) interfere with the interaction of the virus with the cell membrane and block the viral attachment; 3) interact with the viral genomic material, inhibit genome replication, and interrupt cellular factors (e.g., protein synthesis) to inhibit viral replication inside the host cell (Ratan et al., 2021).

Medicinal plants and active ingredients that have already shown antiviral properties against viruses (Wintachai et al., 2015; Sharma et al., 2019), could be further combined with Ag NPs for antiviral treatment. For example, curcumin as a natural polyphenol, which possesses the antiviral activity *via* preventing the replication and budding of virus, has been developed as a reducing agent and capping agent to synthesize curcumin-modified AgNPs (cAgNPs, 11.95 ± 0.23 nm) against respiratory syncytial virus (RSV) infection (Yang et al., 2016). Additionally, based on epigallocatechin gallate (EGCG) that interferes with viral membrane proteins and inhibits the early stages of infection (Carneiro et al., 2016), Saadh et al. conjugated EGCG with AgNPs (EGCG-AgNPs) and found the co-administration with zinc sulfate (for blocking the protease activity, polymerase enzymatic processes, and virus-cell physical processes) (Read et al., 2019) showed much stronger antiviral activities *in vitro* (Saadh and Aldalaen, 2021).

As for other inorganic NPs, SeNPs (Li et al., 2018) and AuNPs (Lysenko et al., 2018) with the low toxicity and great antiviral capabilities have attracted increasing attention in recent years. Furthermore, the metal doping or multicomponent combination has been developed to improve the antiviral activity. For instance, Ag NPs doped titanium dioxide nanopowders (AgNPs/TiO_2_) enhanced the photocatalysis of TiO_2_ by trapping excited electrons to prevent charge recombination, and improved the inactivation rate of bacteriophage MS2 by more than 5 fold than base TiO_2_ through leaching toxic silver ions and increasing the production of hydroxyl radicals (Liga et al., 2011).

### Size

Size-dependent interaction of NPs with virus surface has been reported, which plays a pivotal role in inhibiting the virus from binding to host cells. For instance, Bekele et al. evaluated the antiviral properties of AgNPs (10, 75, and 110 nm) and doses (25, 50, and 100 μg/mL) against feline calicivirus (FCV) as a surrogate for norovirus. The AgNPs_10 nm_ at 50 and 100 μg/mL concentrations inactivated the FCV beyond the limit of detection, prevented the development of cytopathic effects (CPEs), and downregulated expression of the viral capsid protein; while no significant antiviral effect was observed in AgNPs_75 nm_ or AgNPs_110 nm_ (Aschalew et al., 2016). Also, AgNPs with the size of 2 nm and 15 nm instead of 50-100 nm, potently inhibited viral entry step *via* disrupting SARS-CoV-2 viral integrity as revealed by a luciferase-based pseudovirus entry assay. Whereas, 2 nm AgNPs showed a cytotoxicity even at 2 ppm while none of the bigger AgNPs were cytotoxic at this concentration (Jeremiah et al., 2020).

#### Surface Chemistry

The surface chemistry of NPs, including the surface ligands and hydrophobic/hydrophilic characters, highly impacts on the process of virus infection *via* affecting the attachment of virus envelope to cell receptors (Elechiguerra et al., 2005). For instance, Silva et al. focused on the hydrophobic/hydrophilic characters of NPs and studied the surface interactions between silica, cells and viruses *via* synthesizing mSiO_2_ NPs with various polar areas (mSiO_2_-(3-aminopropyl) triethoxysilane (APTES) > mSiO_2_- (3glycidyloxypropyl) trimethoxysilane (GPTMS) > mSiO_2_-tetraethylorthosilicate (TEOS) > mSiO_2_-trimethoxy(2-phenylethyl) silane (TMPES)). They found mSiO_2_-TMPES with higher hydrophobicity would favor the interaction with HIV-gp120 (with a hydrophobic core), while mSiO_2_-APTES with a larger topological polar surface area would be beneficial to interaction with VSV-G envelope (formed by neutral amino acids) (de Souza e Silva et al., 2016). Therefore, the hydrophobic/hydrophilic characters of NPs play a pivotal role in transduction inhibition effect and antiviral activity, and should be considered during the fabrication of antiviral nanosystems.

Therefore, inorganic NPs might be highly potent antivirals against B19V infection. Moreover, they could be combined with small molecule antiviral agents *via* surface modification or coencapsulation.

### Biotherapeutic and Biomimetic Nanosystems

From the perspective of nanomedicine and nanopharmacology, virus-mimetic nanosystems could be designed by mimicking viral surfaces and characteristics for anti-viral therapy (Maslanka Figueroa et al., 2021). It is possible to construct biomimetic nanosystems through modifying B19V capsid (B19V-mimetic) or glylipidside (P antigen) on the surface of the EPCs (EPCs-mimetic), in order to inhibit the binding of B19V capsid to membrane glylipidside and thereby halt the pathogenesis of the viral infection. Moreover, the vaccine nanotechnologies might be also employed to encapsulate genomic material or protein/peptide antigens in NPs, which deliver nucleic acids (mRNA and DNA vaccines) to host cells, and/or antigens (subunit vaccines) to immune cells (Chung et al., 2020).

Additionally, exosome owns the attractive features like cell targeting, low-immunogenicity, safety, and high biocompatibility, thus potentiating their role as an appealing therapeutic agent and/or a drug delivery nano-platform (Pinky et al., 2021). For instance, Sengupta et al. studied exosomes (ExoFlo™) that are derived from allogeneic bone marrow MSCs (bmMSC) for treatment of severe COVID-19 in a prospective nonrandomized open-label cohort study. bmMSC-derived exosomes with a single intravenous dose could reverse hypoxia, downregulate cytokine storm, and reconstitute immunity in severe COVID-19 patients (Vikram et al., 2020). Thus, bmMSC-derived exosomes might also be a promising therapeutic candidate for B19V.

## Conclusion and Outlook

All in all, although several antiviral agents against B19V have been reported, the majority of them are only tested *in vitro* and have a long way to go (involving safety and efficacy tests, as well as cost-effectiveness analyses) from lab to shelf. Apparently, numerous efforts should be made in following years to lay the foundations toward counteracting B19V *via* developing novel therapeutic agents as well as the infection prevention of high risk groups.

Firstly, the precise understanding related to the structural morphology, virus–cell interactions, pathophysiology, and related immunological response of B19V is vital for pharmacology and nanotechnology scientists. Secondly, more antiviral compounds that are already in use for other viruses should be considered and evaluated against B19V. Besides, we should be focused on exploring other targets (e.g. uncoating inhibitors, and protease inhibitors) or modifying the reported antiviral agents (e.g., DNA synthesis inhibitors) to improve the selectivity and effectiveness of anti-B19V treatment. Thirdly, it is equally important to explore a suitable nanocarrier delivery technology (e.g., drug/antigen carriers, delivery technology) to boost the effectiveness and biosafety of the therapeutics or vaccines. Moreover, B19V-tailored functional nanosystems that can be employed in the common noninvasive imaging modalities would gain more prominence for monitoring infectious sides and treatment responses (Wang et al., 2021; Lee et al., 2022).

In this era of advanced bioscience and nanoscience, scientists with incredible diverse backgrounds have converged in fruitful cooperation, playing a frontline role in tackling this outbreak. And the above attempts will offer novel opportunities for exploring more efficient, targeted antiviral agents, which can be translated to available therapeutic options in the near future.

## Author Contributions

KM and XH conceived the idea. XH, KM and CJ wrote about the manuscript. KM, XH, JW, and JZ polished the manuscript. XH, CJ, and ZJ prepared the figures. All authors read, discussed, and approved this manuscript.

## Funding

This work was supported by National Natural Science Foundation of China (Grant number: 32000985, 81900683), Zhejiang Provincial Natural Science Foundation (Grant number: LQ21H300003), Zhejiang Province Medical and Health Science Research Project (Grant number: 2021KY666), Zhejiang Pharmaceutical Association (Grant number: 2019ZYY12), and Gansu Province Science Foundation for Youths (Grant number: 21JR7RA431).

## Conflict of Interest

The authors declare that the research was conducted in the absence of any commercial or financial relationships that could be construed as a potential conflict of interest.

## Publisher’s Note

All claims expressed in this article are solely those of the authors and do not necessarily represent those of their affiliated organizations, or those of the publisher, the editors and the reviewers. Any product that may be evaluated in this article, or claim that may be made by its manufacturer, is not guaranteed or endorsed by the publisher.
